# Supplementary material for: Comparison of Three Glycoproteomic Methods for the Analysis of the Secretome of CHO Cells Treated with 1,3,4-O-Bu3ManNAc
Source: Bioengineering (Basel). 2020 Nov 10;7(4):144. doi: 10.3390/bioengineering7040144 (PMC7712478; doi:10.3390/bioengineering7040144)
Supplement: Supplementary file 1 [file bioengineering-07-00144-s001.zip › bioengineering-958307/Supplemental Figure S1.docx]

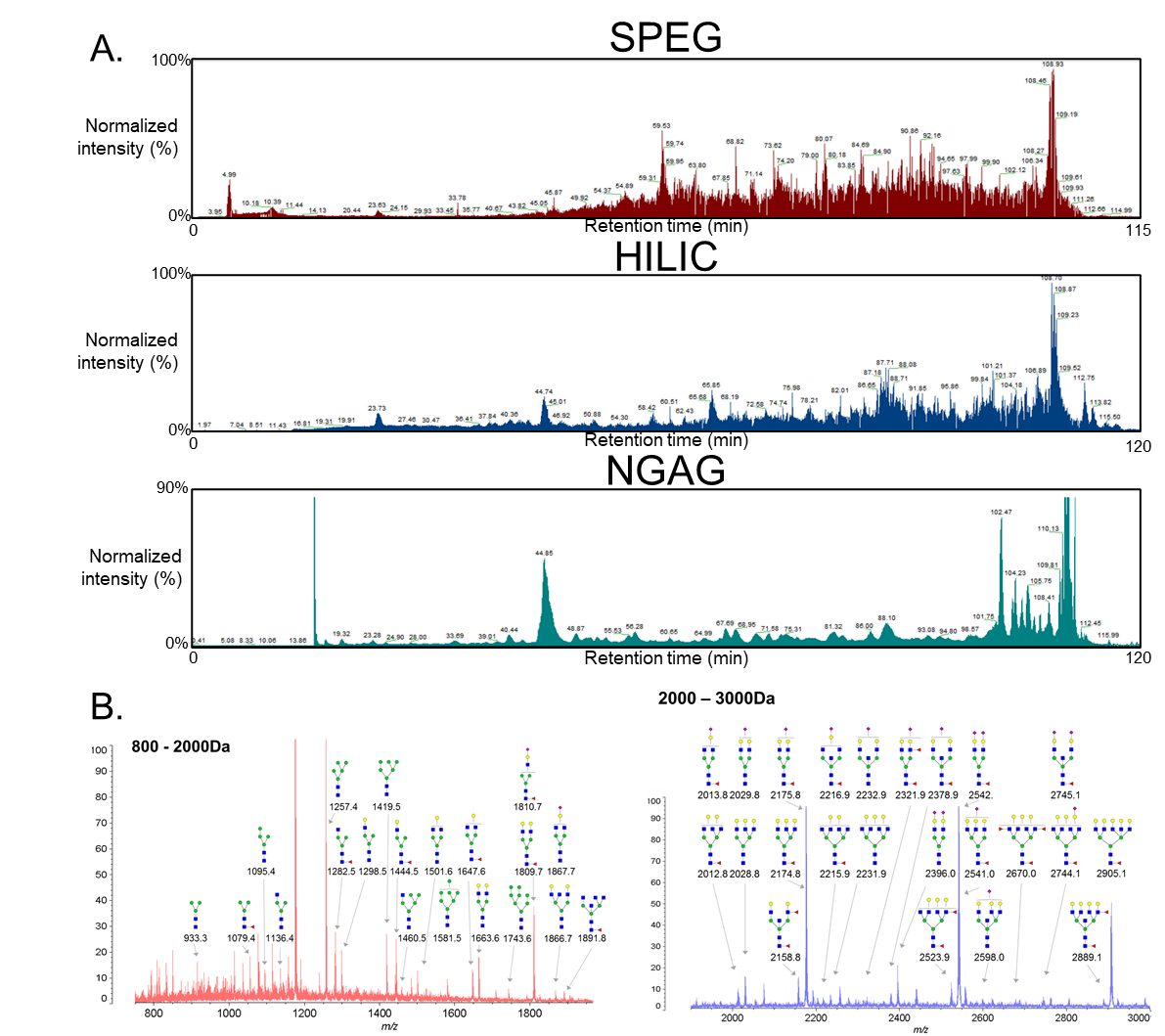


**Supplemental Figure 1.** Gross analysis of the LC-MS spectra. A) Proteome spectra suggest similarity between SPEG and HILIC preparations, with a more distinct trace from NGAG. B) MALDI-TOF glycan analysis traces of glycan samples released by immobilized peptides in the NGAG workflow, normalized to the most intense peak in each range. Searching the identified traces using GlycoPeakFinder identified 60 unique glycans across the range. Representative spectra from the 800-2000 Da and 2000-3000 Da ranges from an untreated control sample presented here. Unlabeled peaks most likely represent glycans with adducts (such as sodiated Hex7 at 1175.5 m/z) and background ions that did not pass filtering in GlycoPeakFinder searches.
